# Disrupted connectivity in schizophrenia: modelling the impact of structural connectivity changes on the dynamics of spontaneous functional networks

**DOI:** 10.1186/1471-2202-14-S1-P100

**Published:** 2013-07-08

**Authors:** Joana Cabral, Henrique Fernandes, Tim Van Hartevelt, Anthony James, Morten L Kringelbach, Gustavo Deco

**Affiliations:** 1 Theoretical and Computational Neuroscience Group, Center of Brain and Cognition, Universitat Pompeu Fabra, Barcelona, 08018, Spain; 2 Department of Psychiatry, University of Oxford, Oxford, OX3 7JX, UK; 3 Warneford Hospital, Oxford, OX3 7JX, UK; 4 Center of Functionally Integrative Neuroscience (CFIN), Aarhus University, Aarhus, Denmark; 5 Institució Catalana de Recerca i Estudis Avançats (ICREA), Barcelona, 08010, Spain

## 

The neuropathology of schizophrenia remains unclear. Some insight has come from modern neuroimaging techniques, which offer an unparalleled opportunity to explore *in vivo *the structure and function of the brain. In particular, a number of studies have found significant alterations in large-scale resting-state functional connectivity (FC) in the disease. The origin of these FC alterations and its potential link with the underlying structure, remain unclear. The FC between brain areas during rest (measured as the temporal correlations of the blood-oxygen-level-dependent (BOLD) signal recorded with functional MRI (fMRI)), is known to be strongly shaped by the underlying structural connectivity. However, the relationship between anatomical and functional brain connectivity is not trivial and computational models of large-scale neural dynamics are unique tools to explore this relationship [1-3]. Importantly, models can be used to predict the effects of structural alterations on the large-scale brain dynamics [4,5], which is beyond reach on the experimental side.

In this work, the structural connectomes from XX patients with schizophrenia and from XX age- and gender-matched controls were built from DTI data using advanced tractography algorithms to detect the white matter tracts between 90 brain areas. In the model, each brain area was represented by a pool of spiking neurons, and its activity was described by a dynamic mean field model. Each brain area -or node in the global network- receives excitatory input from structurally connected regions in proportion to the number of fibre tracts detected, which may vary from subject to subject.

The large-scale spontaneous activity, simulated with the model using the different structural connectomes, was compared between patients and controls. We have found that, in schizophrenia, the coupling weights are weaker, which shifts the bifurcation point (above which the dynamics becomes unstable) to a higher global coupling weight. In addition, the simulated mean field activity was transformed into BOLD signal, and the properties of the simulated FCs were analyzed using measures from graph theory.

Our results indicate that the subtle randomization of functional networks occurring in schizophrenia is related to alterations in the underlying structural connectivity, which shift the dynamical regime of the brain at rest further away from the bifurcation point, which may have an impact on the behavioural symptoms of schizophrenia.
